# Effect of sitagliptin and glimepiride on C-reactive protein (CRP) in overweight Type-2 diabetic patients

**DOI:** 10.12669/pjms.35.2.645

**Published:** 2019

**Authors:** Mazhar Hussain, Muhammad Aamir Rafique, Javed Iqbal, Lubna Akhtar

**Affiliations:** 1 *Dr. Mazhar Hussain, MBBS, M.Phil (Pharmacology). Associate Professor of Pharmacology, Department of Pharmacology, Sheikh Zayed Medical College, Rahim Yar Khan, Punjab, Pakistan*; 2 *Muhammad Aamir Rafique, MBBS, M.Phil (Pharmacology). Assistant Professor of Pharmacology, Department of Pharmacology, Islam Medical, Sialkot, Punjab, Pakistan*; 3 *Dr. Javed Iqbal, MBBS, FCPS (Medicine). Assistant Professor, Department of Medicine, Sheikh Zayed Medical College, Rahim Yar Khan, Punjab, Pakistan*; 4 *Dr. Lubna Akhtar, MBBS, FCPS. (Gynae & Obs). Senior Demonstrator Pharmacology, Department of Pharmacology, Sheikh Zayed Medical College, Rahim Yar Khan, Punjab, Pakistan*

**Keywords:** Type-2 Diabetes, Sitagliptin, Glimepiride, Glycemic index, Lipid profile, C- reactive protein

## Abstract

**Objectives:**

To compare the anti-inflammatory effect of sitagliptin and glimepiride by measuring CRP in overweight Type-2 diabetic patients.

**Methods:**

This clinical trial was conducted at diabetic clinic of Islam Central Hospital, Sialkot over a period of six months from June to November 2017. A total of 110 overweight Type-2 diabetic patients were divided in to two groups. Group-A was given tablet sitagliptin 50mg while Group-B was given tablet glimepiride 2mg for a period of 12 weeks. The dose was titrated according to blood sugar level. The primary outcome was measuring changes in CRP while secondary outcomes was changes in BMI, blood sugar, HbA1C, lipid profile and CRP from baseline in both study group using SPSS 16.

**Results:**

After 12 weeks treatment, body weight increased in glimepiride but slightly reduced in sitagliptin, however comparison between them was non significant (p=0.07). Although both groups reduced blood sugar and HbA1c but comparison between them was non significant (p=0.59 and p=0.17 respectively) value. However lipid profile improved significantly in sitagliptin vs. glimepiride group i.e total cholesterol (-25±32.5 vs +1.5±45.4 P=0.02) triglycerides (-19±44.6 vs-1.8±48.7 P=0.001) LDL- cholesterol (-10±22.4 vs-0.8±18.7 P=0.001) HDL-cholesterol (-2.6±6.2 vs 1.2±5.2 P=0.03).Sitagliptin significantly reduced CRP in comparison to glimepiride (-2.3±1.8 vs0.8±1.5 P=0.001).

**Conclusion:**

Sitagliptin has strong anti inflammatory effect marked by reduction in CRP level in comparison to glimepiride in overweight type-2 diabetic patients. It also exerted beneficial effect on glycemic and lipid profiles.

## INTRODUCTION

Inflammation always play a dominant role in initiation, progression as well complication of multiple cardiovascular disorders in diabetic patients.^1^C-reactive protein (CRP) is one of the most potential and studied inflammatory marker in various cardiovascular as well as non cardiovascular disorders. C-reactive protein (CRP) detects ongoing inflammation as an acute phase reactant. It not only identifies inflammation in joints but also detects severity of inflammation in various cardiovascular diseases. It is considered to be one of the independent as well as combined risk factor in a number of cardiovascular disorders.^2^ CRP causes the endothelial cells to generate monocyte chemotactic protein-1, which can lead to increase expression of intercellular adhesion molecule-1 and vascular adhesion molecule-1. Moreover, CRP induces the monocyte cells to release pro-inflammatory cytokines which play a major role in the pathogenesis of cardiovascular disease.^3^CRP is measured in various cardiac and vascular diseases associated experimental as well as clinical trials. Studies also revealed the possible role of CRP in assessment of fluctuations in lipid profile in response to dietary intake. They pointed out that these changes depend on the individual’s baseline CRP concentration. Individuals with high baseline CRP has deranged serum lipid profile and vice versa. Similarly after consuming high fat diet, there is increase in the baseline CRP, cholesterol, triglycerides and LDL-cholesterol while consuming low fat diet has opposite effects. Obese patients also have increase baseline CRP as compared to their lean counterparts.^4^

Sitagliptin is one of the oral anti diabetic drug that inhibits enzyme DPP-4.DPP-4 increases the physiological concentration of GLP and GIP in body. Theses enzymes have Pleiotropic effects on body such as control of blood sugar, dyslipidemia, hypertension, oxidative stress and silent inflammation. Studies have shown that sitagliptin produce anti inflammatory effects by reducing CRP in both diabetic as well as non diabetics.^5^-^6^

Keeping in view these studies there is need for thorough investigation to determine anti-inflammatory effect of sitagliptin. Hence the present study was conducted to determine the effect of sitagliptin and glimepiride on CRP in overweight Type-2 diabetic patients.

## METHODS

This clinical trial was started after taking approval from ethical committee of Islam Medical College Sialkot. A verbal consent was taken and perspectives of study were clearly explained to patients before the start of study. Overall 450 Type-2 diabetic patients taking oral antidiabetic agents were screened over a period of six months from June to November 2017 at diabetic clinic. From these 110 patients were enrolled in the study on the basis of following criteria. 110 Type-2 diabetic patients having HbA1C < 9, BMI>25, taking glimepiride as an oral antidiabetic agent were included in the study. Patients with history of pregnancy, alcohol, smoking, BMI>30, HbA1C>10 and Type-1 diabetes were excluded from the study. Patients with history of ischemic heart disease, hypertension, heart failure, liver and renal disorders and those who were taking any drugs that can affect inflammation such as aspirin, NSAIDS, steroids, metformin, pioglitazone, DPP-4 inhibitors, anti platelets, ACE inhibitors, Vitamin E, beta blockers, statins and other lipid lowering drugs were excluded from the study. These patients were randomly divided 1:1 ratio in stratified block and computerized number is allocated in chronological order to each patients design by computed-generated software. Patients in Group-A were switched from glimepiride 2mg to take oral Sitagliptin 50mg after a wash period of one week for a period of 12 weeks while patients in Group-B continued tab glimepiride 2mg for whole study period. The doses of the both tablets were titrated according to their blood sugar level. Body weight was measured by wearing light clothes by digital weight machine. Height was measured without wearing shoes by microtoise. BMI was calculated by using standard formula weight in kg divided by height in meter square (kg/m^2^) Blood sample was taken after an overnight fasting of 12 hours by cephalic vein puncture. After clotting and centrifugation, serum was separated and stored at4 °C for one hour until analyzed for C - reactive protein. CRP in each sample was assayed by an immunoturbidimetric method by using a standard assay kit. Blood sugar level was estimated by oxidase method. Liquid chromatography and enzymatic end point method was used to determined HbA1c and serum lipid profile respectively.

### Data Analysis

A sample size (45 per group) was calculated to detect a difference of HbA1c level over 0.5% with 90% power and 5% significance. The sample size was increased to 55 per group to accommodate anticipated dropout rate. Data was analyzed by SPSS 16. Fisher’s exact test was used for comparison of non parametric data in terms of sex & duration of disease was expressed as numbers and percentiles. Parametric data in terms of body weight, BMI, blood sugar, HbAIc and lipid profiles were expressed as mean± SD from baseline .This comparison was done by paired t test. P value <0.05 were seemed to be statistically significant. Unpaired t-test was used to compare Group-A & B at 12 weeks.

## RESULTS

The safety and tolerability profile of both drugs were good during the study period. Out of 110 patients, 08 patients failed to complete the study. Five patients in glimepiride group developed hypoglycemia while three patients in sitagliptin failed to follow up during the study. Overall 102 patients completed the study 52 in glimepiride Group-And 50 in sitagliptin group. The baseline demographic and laboratory data of both groups is shown in Table-I. After 12 weeks treatment, although body weight increased in glimepiride but slightly reduced in sitagliptin and comparison between them was non significant (p=0.07). Similarly comparison in terms of blood sugar and HbA1c in both groups were also non significant (p=0.59 and p=0.17 respectively) value. Lipid profile improved significantly in sitagliptin vs. glimepiride i.e total cholesterol (25±32.5 vs +1.5±45.4 P=0.02) triglycerides (-19±44.6 vs-1.8±48.7 P=0.001) LDL- cholesterol (-10±22.4 vs-0.8±18.7 P=0.001) HDL-cholesterol (-2.6±6.2 vs 1.2±5.2 P=0.03).Sitagliptin also significantly reduced CRP in comparison to glimepiride (-2.3±1.8 vs0.8±1.5 P=0.001).These results are shown in Table-II.

**Table-I:** Baseline demographic characteristics of both study groups.

Demographic Characteristics	Number of Patients (N=110)
Age (years) (mean ±SD)	54±8
Sex (male/female)	70(63.6%)/40(36.3%)
BMI (kg/m^2^) (mean ± SD)	27±4
Duration of Tyupe-2 diabetes mellitus (years) (mean±SD)	6.8±2.5
*Laboratory parameters (mean ± SD)*	
Fasting Blood glucose(mg/dl)	156±10.5
HbA1c (%)	7.9±2.8
Total Cholesterol(mg/dl)	192±23.5
Triglycerides(mg/dl)	187±19.2
LDL-Cholesterol(mg/dl)	120±12.4
HDL-Cholesterol(mg/dl)	41.3±3.6
hsCRP (mg/L)	5.8±6.7

**Table-II:** Mean Changes of study parameters from baseline in both study groups.

Parameters	Glimepiride (N=52)	Sitagliptin (N=50)	P value
BMI (kg/m^2^)	+0.7±0.9	-0.02±0.7	0.07
Fasting Blood glucose (mg/dl)	-35±14.2	-27±18.4	0.59
HbA1c (%)	-2.4±4.8	-1.8±5.2	0.17
Total Cholesterol (mg/dl)	+1.5±45.4	-25±32.5	0.02
Triglycerides (mg/dl)	-1.8±48.7	-19±44.6	0.001
LDL-Cholesterol (mg/dl)	-0.8±18.7	-10±22.4	0.04
HDL-Cholesterol (mg/dl)	1.2±5.2	-2.6±6.2	0.03
hsCRP (mg/L)	0.8±1.5	-2.3±1.8	0.001

## DISCUSSION

Inflammation that cannot manifests clinically is called silent or subclinical inflammation. Identification of this silent inflammation is crucial in obese and diabetic patients as it can lead to significant morbidity and mortality. This poses an enormous burden clinically as well as economically on health system.^7^ In order to detect this inflammation there are several inflammatory markers but CRP is considered to be the one of the most studied inflammatory marker because of its uniformity, stability and long half-life. It plays an important role in detecting inflammation, asses the fitness level of general population and act as a disease and drug monitoring tool.^8^,^9^

After 12 weeks treatment, sitagliptin reduce inflammation marked by the reduction of CRP. This reduction in CRP was further accompanied by improvement in glycemic control and lipid profile but neutral effect on body weight.

Results of our secondary objective in terms of glycemic control, body weight and lipid profile were also similar to various other studies. These studies demonstrated that sitagliptin is considered to be one of the safest oral antidiabetic drugs. Diabetic’s patients tolerated it very well with adequate glycemic control without risk of hypoglycemia. Sitagliptin has neutral or mild weight reducing effect. Moreover it has beneficial effects on serum lipid profile either alone or in combination. Findings in these studies were in accordance with our study.^10^-^13^

The results of our primary objective regarding inflammation were consistent with study conducted by Asaharaetal^14^ who revealed that sitagliptin50mg daily for a period of three months not only improved glycemic control, lipid profiles but also reduced inflammatory markers level such as CRP and TNF-α in Type-2 diabetic patients. Similar type of study was conducted by Tremblay & colleagues^15^ concluded that after just six weeks treatment with sitagliptin, it has more pronounced effect on elevated CRP and cell adhesions level as compared to normal values in Type-2 diabetic patients. This effect represented that sitagliptin may be very beneficial in those Type-2 diabetic patients that have proatherogenic comorbidities. In another study sitagliptinin addition to its glycemic control effect significantly improved endothelial dysfunction and inflammation in patients who have uncontrolled diabetes and coronary artery disease.^16^

A study conducted by Makdissi et al^6^ pointed out that sitagliptin not only reduced CRP and IL-6 level but also has strong anti-atherogenic properties. It inhibits inflammatory signaling mechanism by reducing the expression of CD26, toll like receptor (TLR-2 &TLR-R), mitogen-activated protein kinase(MAPK-8), chemokine receptor 2(CCR-2), nuclear factor Kappa-B kinase and TNF-α on mononuclear cells. Sitagliptin also reduced epicardial adipose tissue thickness (EAT) significantly which is a marker of visceral fat in obese Tyupe-2 diabetic patients who were inadequately controlled on metformin monotherapy.^17^

All these above mentioned studies have strongly indicated that cardiovascular protection by sitagliptin involve multiple molecular and cellular mechanism like reduction in oxidative stress, silent inflammation, cell apoptosis and tissue repair. However on the contrary there were few studies which revealed that although sitagliptin significantly improved hemodynamic and metabolic parameters in Tyupe-2 diabetic patients but failed to improved inflammation marked by reduction of CRP.^18^-^20^ The probable reason in these studies was that some of the patients have already concurrent illness such as dyslipidemia, obesity, hypertension and coronary artery disease. Some patients were smoking and drinking too. All these conditions has strong effects on inflammation and its marker c-reactive protein. This may confounded the results in these studies

### Future Recommendation

Seeing these paradoxical effects of sitagliptin there is further need to explore its effect on inflammation in Tyupe-2 diabetic patients. For this reason, future clinical studies of larger sample size are needed to solve this ambiguity. Moreover other inflammatory and oxidative markers would be taken into account in order to explore its cardio protective effects.

### Limitation of Study

We did not check other inflammatory markers like TNF-α and various interleukins due to cost.

## CONCLUSION

Sitagliptin has strong anti inflammatory effect marked by reduction in CRP level in comparison to glimepiride in overweight type-2 diabetic patients. It also exerted beneficial effect on glycemic and lipid profiles.
